# hsa_circ_0010889 downregulation inhibits malignant glioma progression by modulating the miR-590-5p/SATB1 axis

**DOI:** 10.18632/aging.204907

**Published:** 2023-08-03

**Authors:** Zhi Wu, Quanlin Guan

**Affiliations:** 1 Department of Neurosurgery, The First Hospital of Lanzhou University, Lanzhou, Gansu 730000, China; 2 Department of Oncological Surgery, The First Hospital of Lanzhou University, Lanzhou, Gansu 730000, China

**Keywords:** glioma, hsa_circ_0010889, miR-590-5p, SATB1, malignant progression

## Abstract

Glioma is a general neurological tumor and circular RNAs (circRNAs) have been implicated in glioma development. However, the underlying mechanisms and circRNA biological functions responsible for the regulation of glioma progression remain unknown. In this study, we employ next-generation sequencing (NGS) to investigate altered circRNA expression in glioma tissues. Regulatory mechanisms were studied using luciferase reporter analyses, transwell migration, CCK8, and EdU analysis. Tumorigenesis and metastasis assays were utilized to determine the function of hsa_circ_0010889 in glioma. Our results showed that hsa_circ_0010889 expression increased in glioma cell lines and tissues, indicating that hsa_circ_0010889 may be involved in glioma progression. Downregulation of hsa_circ_0010889 inhibited glioma invasion and proliferation in both *in vitro* and *in vivo* experiments and luciferase report assays found that miR-590-5p and SATB1 were downstream targets for hsa_circ_0010889. SATB1 overexpression or miR-590-5p inhibition reversed glioma cells proliferation and migration post-silencing of hsa_circ_0010889. Taken together, our study demonstrates that hsa_circ_0010889 downregulation inhibits glioma progression through the miR-590-5p/SATB1 axis.

## INTRODUCTION

Glioma is a major primary brain tumor, resulting in ~75% of malignant central nervous system (CNS) cancers among adults [1, 2]. Currently, resection surgery and temozolomide (TMZ)-based chemotherapy and radiotherapy are the main treatment methods for glioma. Nevertheless, multidrug resistance, recurrence, and metastasis are often important factors associated with poor clinical efficacy. Therefore, glioma remains an incurable malignancy with a mean survival duration of around 12–15 months [3, 4]. Therefore, it is essential to identify unknown pathogenic mechanisms associated with glioma progression and to identify diagnostic markers and precise therapeutic targets.

Circular RNAs (circRNAs) is a type of single-stranded noncoding RNA having covalently closed-loop structures, which are considered as promising biomarkers and targets for the diagnosis and treatment of many diseases, particularly cancer. Accumulating evidence suggests that EWSR1-induced circNEIL3/IGF2BP3 enhances glioma progression by regulating macrophage polarization [5] and circRNA-0002109 enhances glioma malignant progresses by regulating the miR-129-5P/EMP2 axis [6]. Exosomal circRNA_104948 promotes glioma progression via the regulation of miR-29b-3p/DNMT3B/MTSS1 signaling [7]. However, the role of circRNA in glioma progression remains largely unclear.

The present study found that hsa_circ_0010889 expression increased in glioma cell lines and tissues. Downregulation of hsa_circ_0010889 inhibited glioma progression in both *in vitro* and *in vivo* experiments. Luciferase reporter assay results showed that miR-590-5p and SATB1 were downstream targets for hsa_circ_0010889. The aim of the present study was to discuss the regulation mechanism of hsa_circ_0010889 in glioma.

## MATERIALS AND METHODS

### Animal use and ethical statement

Nude BALB/c female mice (four weeks old, 15–20 g) were obtained from SLAC Laboratory Animal Co. Ltd., Shanghai, China. They were housed in independently ventilated cages and kept at 24–26°C, with constant humidity and a 12-h light/dark cycle. The Ethics Committee of The First Hospital of Lanzhou University oversaw all the procedures (LDYYLL2021-23).

### RNA sequencing, quantification, and identification of circRNAs

Total RNA was extracted from pairs of freshly frozen glioma and adjacent tissues. An Agilent 2200 system (Agilent Technologies, Santa Clara, CA, USA) was used to confirm the RNA quality. The RiboMinus eukaryote kit (QIAGEN, Valencia, CA, USA) was used to remove ribosomal RNA followed by cDNA library construction. NGS was carried out using the Illumina HiSeq 3000 (Illumina, San Diego, CA, USA) and the reads were aligned to the GRCH37.p13 reference. Unmapped reads were collected to characterize circRNAs. The reads were counted and used for mapping to the circRNA junction with an overhang of ≥6 nt for each candidate.

### Cell culture and transfection

Human glioma cell lines LN229, SHG44, U251, and T98G along with normal glial HEB cell lines were purchased from the Chinese Academy of Sciences Cell Bank (Shanghai, China). We cultured HEB cells in RPMI-1640 medium and cultured Glioma cell lines in DMEM medium supplied with 10% FBS and 1% Penicillin-Streptomycin Solution (Gibco, USA). The cells were maintained in an incubator at 37°C with 5% CO_2_.

SATB1 overexpression vector was constructed by inserting SATB1 cDNA into the pcDNA3.1 vector. Then, *miR-590-5p* mimics and hsa_circ_0010889 siRNA were synthesized by Genepharma (Suzhou, China). Cell transfection was performed at 70% confluency according to Lipofectamine 2000 manufacturer’s instructions. After two days, the cells were harvested for downstream experiments.

### Fluorescence *in situ* hybridization (FISH)

Specific probes for hsa_circ_0010889 (Dig-5′-CTTGCCAGACTTAAGCTTTTTACGACGCG-3′-Dig) were synthesized (Geneseed Biotech, Guangzhou, China) and signals were captured via Cy3-conjugated anti-biotin antibodies (GenePharma, Shanghai, China). 4,6-diamidino-2-phenylindole (DAPI) was also utilized to counterstain for cell nuclei. Finally, we imaged the cells using a Zeiss LSM 700 confocal microscope (Carl Zeiss, Oberkochen, Germany).

### Western blot assay

Protein samples were extracted from cells with RIPA lysis buffer for western blot assays. Anti-E-cadherin (1:1,000), anti-N-cadherin (1:1,000), and anti-GAPDH (1:1,000) primary antibodies were obtained from Cell Signaling Technology (Beverly, MA, USA) and used to stain protein blots following the manufacturer’s instructions. Immunoreactivity was visualized using a chemiluminescence detection kit (ECL; Western Blotting Substrate, Donghuan Biotech, Dongguan, China).

### Quantitative real-time polymerase chain reaction (RT-qPCR)

Total cellular RNA was extracted using a TRIzol reagent kit (Invitrogen, Carlsbad, CA, USA) and cDNA was synthesized for subsequent qPCR using a TaqMan Assay Kit (Applied Biosystems, Foster City, CA, USA) and the 2^−ΔΔCT^ approach was used to obtain relative expression fold changes. *U6* and *GAPDH* were employed as internal references. The following primers were used: hsa_circ_0010889 primers forward, 5′-CCTAATAAATCCTTGC-3′ and reverse, 5′-CAGCTCCGGCAACTAAGCGCGC-3′. *miR-590-5p* primers forward, 5′-GAGCTTATTCATAAAAGT-3′; and reverse: 5′-TCCACGACACGCACTGGATACGAC-3′. U6 primers were forward, 5′-CTCGCTTCGGCAGCACA-3′; and reverse: 5′-AACGCTTCACGAATTTGCGT-3′; GAPDH primers were forward, 5′-AATGGGCAGCCGTTAGGAAA-3′; and reverse: 5′-TGAAGGGGTCATTGATGGCA-3′.

### 5-Ethynyl-2′-deoxyuridine (EdU) assay

DNA synthesis and cell proliferation were analyzed using an EdU assay kit (RiboBio, Guangzhou, China). Here, 1 × 10^4^ of LN229 and U251 cells were seeded into 96-well plates overnight. On the second day, EdU solution (25 μM) was added to the wells and incubated for 24 h. Then, 4% formalin was utilized to fix the cells for 2 h at room temperature. We utilized Triton X-100 to permeabilize the cells for ten minutes, and then added 200 μL Apollo reaction solution to stain EdU and 200 μL of DAPI solution to stain cell nuclei for 0.5 h. DNA synthesis and cell proliferation were measured using a fluorescence microscope (Nikon, Tokyo, Japan).

### Cell proliferation assay

We seeded 2 × 10^3^ cells into 96-well plates and the absorbance at 450 nm was read for every sample using the CCK-8 assay (Yeasen Biotech Co., Ltd., Shanghai, China). Finally, we constructed a cell viability curve.

### Transwell migration assay

Transfected cells after 48 h were diluted 2.0 × 10^5^/mL and briefly, 200 μL/well of cell suspension was added to the Transwell chamber (Millipore, Billerica, MA, USA) upper side. At the same time, we added 500 μL of medium containing 10% FBS to the lower chamber. After a 24 h incubation, we fixed the cells that had migrated to the bottom side with paraformaldehyde for 15 min, and then stained them with crystal violet for 5 mins. Cells were observed under a microscope and the migration cell numbers were counted. We randomly selected and counted five fields of view for every sample.

### Dual-luciferase reporter assay

Putative miR-590-5p binding site in the 3′-UTR for the target gene SATB1 and hsa_circ_0010889 (Mut/WT) were cloned into the psi-CHECK (Promega, Madison, WI, USA) vector downstream of firefly luciferase 3′-UTR or hsa_circ_0010889. The primary luciferase signal was normalized to Renilla luciferase as the normalization signal. The relative Renilla luciferase activity was analyzed according to the provided protocols (Promega, Mannheim, Germany).

### *In vivo* experiments

To establish the nude mouse model for glioma, LN229 cells (1 × 10^6^) with sh-NC or sh-hsa_circ_0010889 were injected into the flank of nude mice. We then measured tumor volume and weight.

For tumor metastasis experiments, luminescence-labeled LN229 cells transfected with sh-NC or sh-hsa_circ_0010889 (1 × 10^5^) were injected into each nude mouse tail vein and post 4 weeks, lung metastasis was assessed using an *in vivo* bioluminescence imaging system and computed metastatic foci counts in lung tissues post H&E staining.

### Statistical analysis

Data were represented as means ± standard deviation (SD) and statistical analyses were performed using GraphPad Prism (La Jolla, CA, USA) to determine statistical significance between groups. A *P*-value of ≤0.05 was considered to be statistically significant. A two-tailed Student’s *t*-test was used to determine significant differences between two groups, and one-way ANOVA with post hoc Bonferroni test was applied to identify significant differences among three or more groups.

### Availability of data and materials

The datasets used and/or analyzed during the present study are available from the corresponding author on reasonable request.

## RESULTS

### A key role for hsa_circ_0010889 in glioma progression

**Figure 1:**
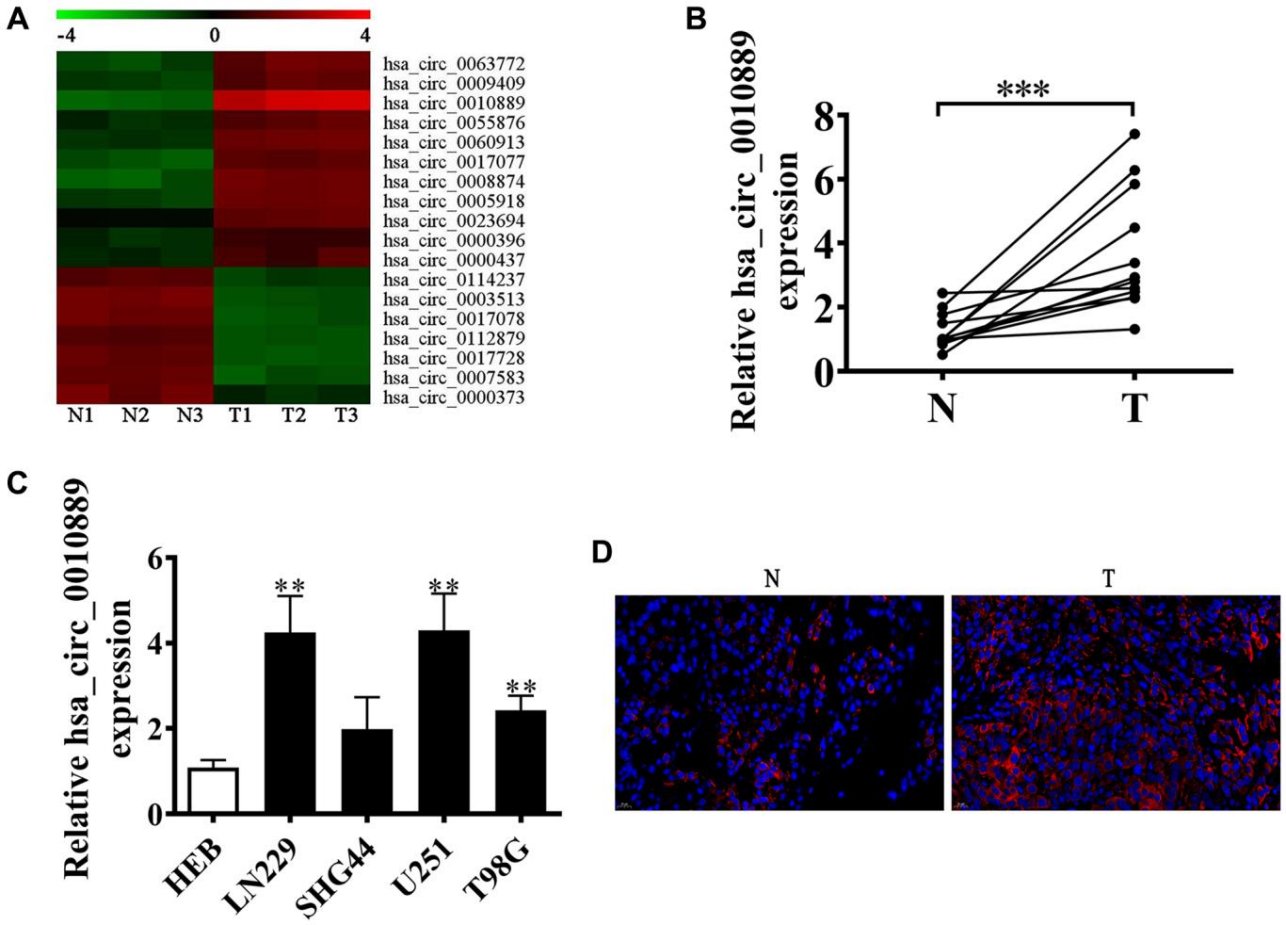
**hsa_circ_0010889 plays a key role in the progression of glioma.** (**A**) Heat map showing the expression of circRNA in CRC tumor tissues and adjacent normal tissues. (**B**) RT-qPCR detection shows the expression of five highly expressed circRNAs in glioma tumor tissues and adjacent normal tissues. The data are presented as the mean ± SD. ^***^p < 0.001 vs. Normal. (**C**) RT-qPCR detection showing the expression of hsa_circ_0010889 in the glioma cell lines LN229, SHG44, U251, and T98G and the normal glial cell line HEB. The data are presented as the mean ± SD. ^**^p < 0.01 vs. HEB. (**D**) FISH detection showing the expression and subcellular distribution of hsa_circ_0010889.

Accumulating evidence suggests that circRNA has an important function in glioma progression [8, 9]. However, its regulatory mechanism is unknown. In our current investigation, we employed next-generation sequencing (NGS) and found that circRNA displayed abnormal expression in glioma tissues when compared to adjacent normal tissues (Figure 1A). Furthermore, RT-qPCR showed high levels of circRNA hsa_circ_0010889 expressions as detected by NGS. Our data show that hsa_circ_0010889 expression was significantly upregulated in glioma tissues (Figure 1B) and RT-qPCR showed that circ-PITHD1 expression increased in several glioma cell lines (SHG44, U251, T98G and LN229) when compared to normal glial HEB cell lines. U251 and LN229 cells have higher hsa_circ_0010889 expression (Figure 1C). FISH data showed that hsa_circ_0010889 expression increased in glioma tumor tissues when compared to adjacent normal tissues (Figure 1D).

### hsa_circ_0010889 downregulation suppressed glioma growth and proliferation *in vivo* and *in vitro*

**Figure 2:**
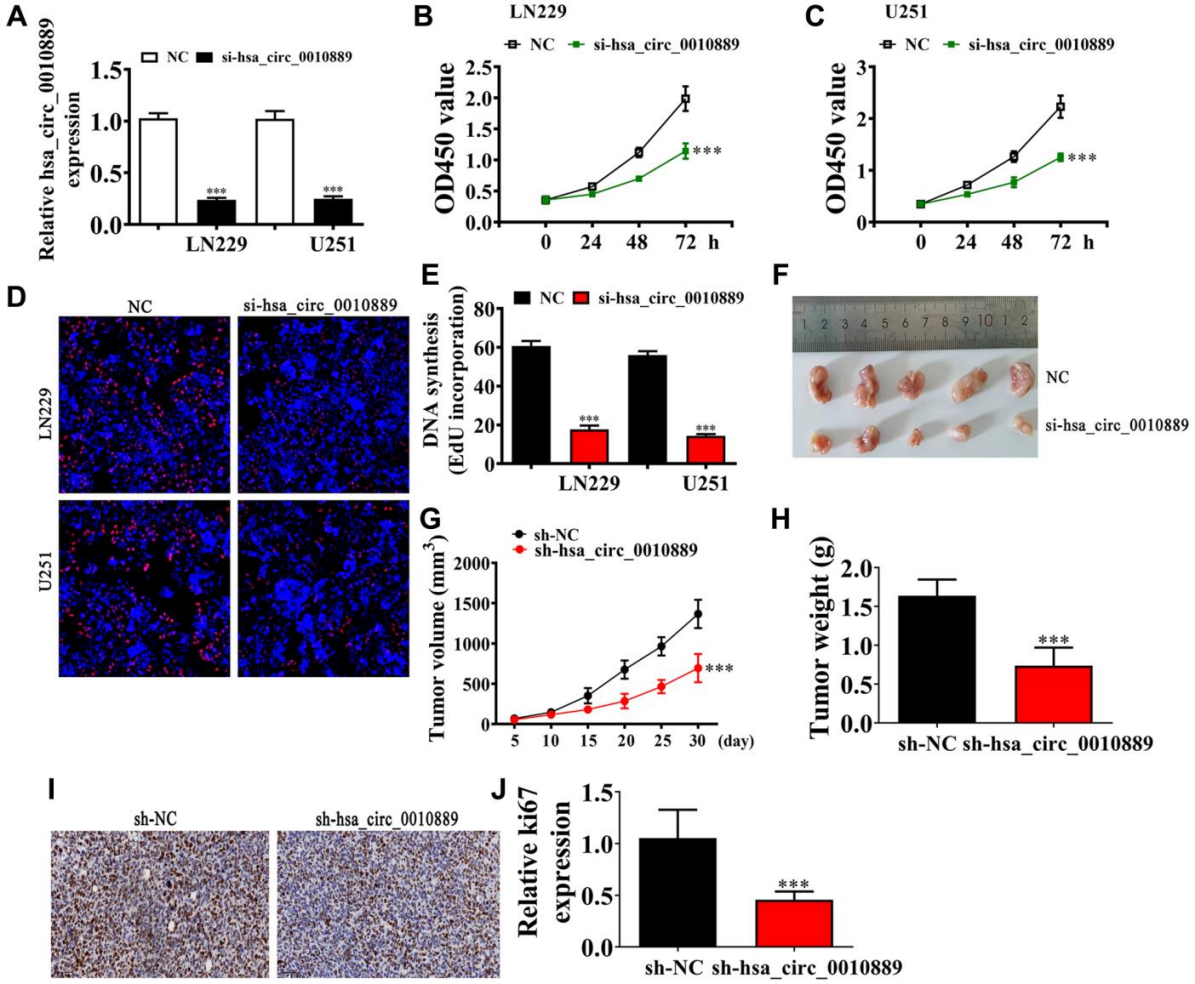
**Downregulation of hsa_circ_0010889 suppressed glioma proliferation and tumor growth both *in vivo* and *in vitro*.** (**A**) RT-qPCR detection showing the expression of hsa_circ_0010889 in both LN229 and U251 cells. The data are presented as the mean ± SD. ^***^p < 0.001 vs. NC. (**B**, **C**) CCK8 detection showing the effect of hsa_circ_0010889 on glioma cell proliferation. The data are presented as the mean ± SD. ^***^p < 0.001 vs. NC. (**D**, **E**) EdU detection showing cell proliferation in both LN229 and U251 cells. Data are presented as the mean ± SD. ^***^P < 0.001 vs. si-NC. (**F**) Representative images of LN229 tumor formation in xenografts from nude mice. (**G**, **H**) Summary of tumor volumes and weights in mice. Data are presented as the mean ± SD. ^***^P < 0.001 vs. sh-NC. (**I**, **J**) Immunohistochemical staining showing the percentage of Ki-67-positive cells and relative Ki-67-positive cells were calculated. Data are presented as the mean ± SD. ^***^P < 0.001 vs. sh-NC.

To determine a role for hsa_circ_0010889 in glioma progression, we constructed an siRNA against hsa_circ_0010889 (si-hsa_circ_0010889), and transfected this into both LN229 and U251 cells. Results showed that hsa_circ_0010889 significantly decreased post hsa_circ_0010889 silencing in U251 and LN229 cells (Figure 2A). Our CCK8 (Figure 2B, 2C) and EdU (Figure 2D, 2E) assays showed that downregulation of hsa_circ_0010889 significantly decreased the proliferation ability of both LN229 and U251 cells. The tumor progression in xenografted nude mice using LN229 cells showed that silencing hsa_circ_0010889 significantly decreased tumor growth in both weight and volume (Figure 2F–2H). Immunohistochemical staining for Ki67 confirmed that hsa_circ_0010889 silencing inhibited Ki67 expression in tumor tissues (Figure 2I, 2J) implying that hsa_circ_0010889 downregulation inhibited glioma proliferation and tumor growth *in vitro* and *in vivo* experiments.

### hsa_circ_0010889 downregulation inhibited glioma pulmonary metastasis and migration *in vitro* and *in vivo*

**Figure 3:**
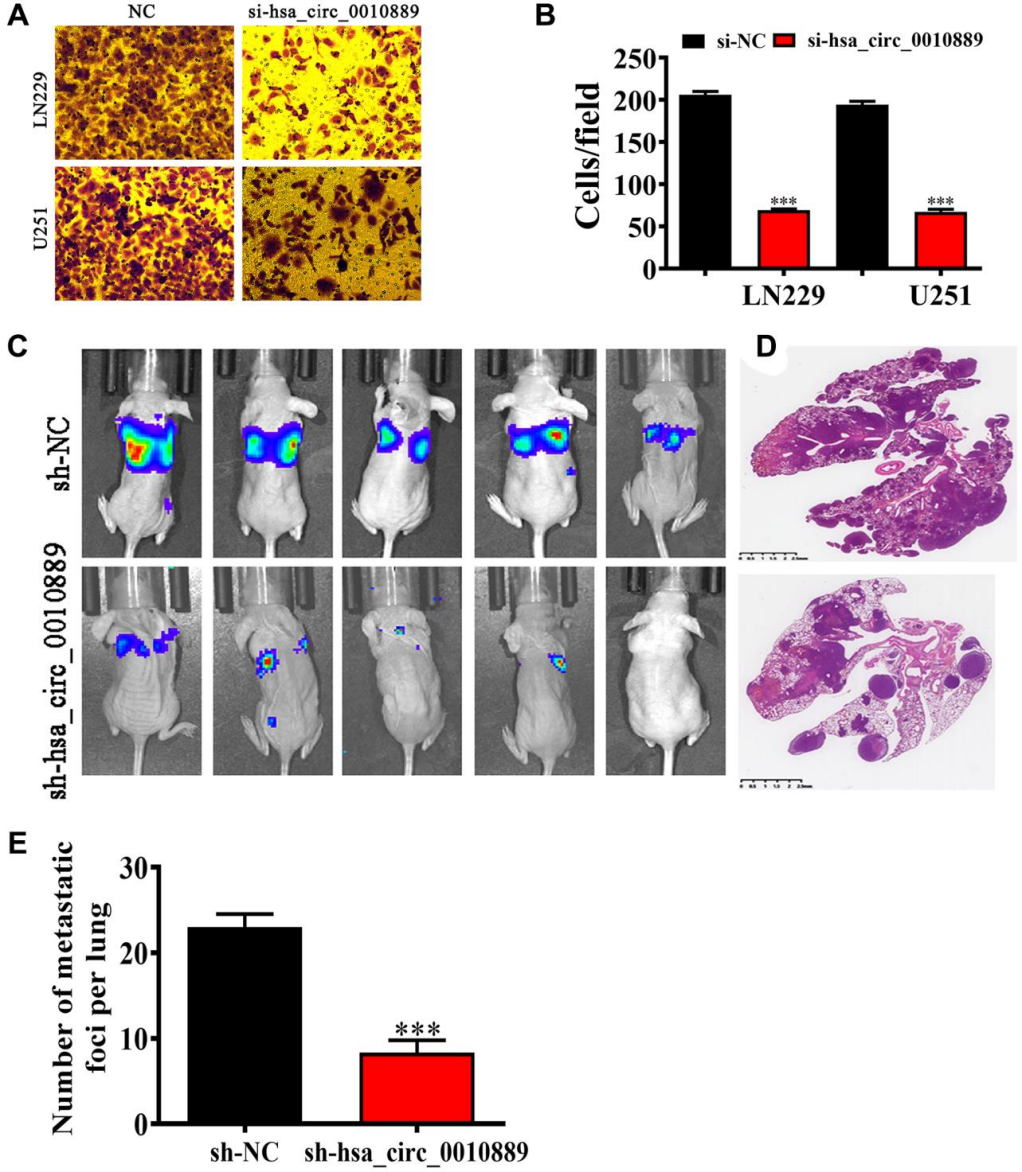
**Downregulation of hsa_circ_0010889 suppressed glioma migration and pulmonary metastasis both *in vivo* and *in vitro*.** (**A**, **B**) Transwell detection showing the migration of both LN229 and U251 cells after transfection with si-hsa_circ_0010889. Data are presented as the mean ± SD. ^***^P < 0.001 vs. si-NC. (**C**) Live image detection showing LN229 cell pulmonary metastasis. (**D**, **E**) The numbers of metastatic foci in lung tissues were calculated according to the H&E staining. The data are expressed as the mean ± SD. ^***^p < 0.001 vs. sh-NC.

Transwell assays looking at migration showed that hsa_circ_0010889 silencing inhibited migration in both LN229 and U251 cells (Figure 3A, 3B) and live imaging data revealed that LN229 cells metastasized to the pulmonary system and that hsa_circ_0010889 silencing decreases this pulmonary metastatic capability. H&E staining also confirmed that hsa_circ_0010889 silencing reduced metastatic foci count in lung tissues (Figure 3C–3E) suggesting that hsa_circ_0010889 downregulation inhibited glioma cells invasion.

### SATB1 and miR-590-5p are downstream targets for hsa_circ_0010889

**Figure 4:**
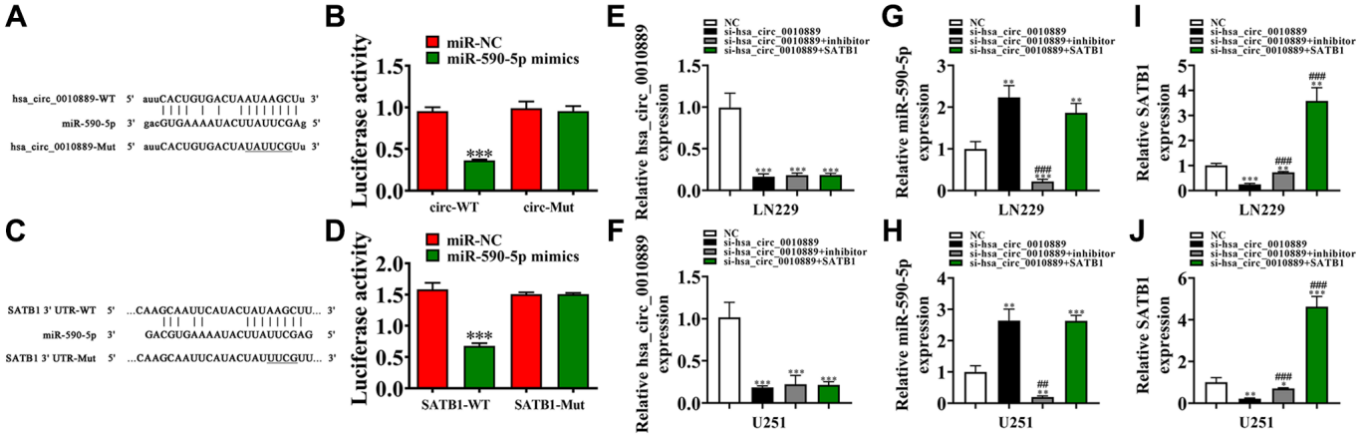
**Both miR-590-5p and SATB1 were the downstream targets for hsa_circ_0010889.** (**A**) Prediction of binding sites of miR-590-5p in hsa_circ_0010889. The MUT version of hsa_circ_0010889 is presented. (**B**) Relative luciferase activity determined 48 h after transfection of LN229 cells with miR-590-5p mimic/NC or hsa_circ_0010889 WT/Mut. Data are presented as means ± SD. ^***^P < 0.001. (**C**) Prediction of binding sites of miR-590-5p within the 3′UTR of SATB1. The MUT version of the 3′-UTR-SATB1 is shown. (**D**) Relative luciferase activity determined 48 h after transfection of LN229 cells with miR-590-5p mimic/NC or 3′UTR-SATB1 WT/Mut. Data are presented as means ± SD. ^***^P < 0.001. (**E**–**J**) RT-qPCR detection showing the expression of hsa_circ_0010889, miR-590-5p and SATB1 in both LN229 and U251 after transfection with si-hsa_circ_0010889, miR-590-5p inhibitor and SATB1 overexpression vector together or single. Data are presented as means ± SD. ^*^P < 0.05, ^**^P < 0.01, ^***^P < 0.001 vs. NC. ^##^*P* < 0.01, ^###^*P* < 0.001 vs. si-hsa_circ_0010889.

Bioinformatics results found that hsa_circ_0010889 interacted with miR-590-5p. A luciferase reporter analysis further confirmed that miR-590-5p inhibited luciferase function in WT cells (Figure 4A, 4B), suggesting that miR-590-5p was the downstream target of hsa_circ_0010889.

Bioinformatics data also found that SATB1 was the miR-590-5p downstream target. Therefore, to better validate the association between SATB1 and miR-590-5p, WT/MUT 3′UTR-SATB1 sequences including a miR-590-5p binding sequence were constructed into a luciferase reporter vector (Figure 4C), which we transfected into LN229 cells integrated in the presence or absence of a miR-590-5p mimic. Luciferase reporter results confirmed that miR-590-5p suppressed luciferase function in WT cells (Figure 4D), showing that SATB1 was the downstream target for miR-590-5p.

Furthermore, RT-qPCR results showed that hsa_circ_0010889 expression decreased post transfected with hsa_circ_0010889 silencing vector. But a miR-590-5p inhibitor or overexpression of SATB1 had no effect on hsa_circ_0010889 expression in U251 and LN229 cells (Figure 4E, 4F), suggesting that SATB1 and miR-590-5p were downstream targets of hsa_circ_0010889. RT-qPCR data also found that hsa_circ_0010889 silencing increased miR-590-5p expression and that SATB1 overexpression had no effect on si-hsa_circ_0010889-induced miR-590-5p expression inhibition (Figure 4G, 4H), suggesting that miR-590-5p was downstream of hsa_circ_0010889. Results also showed that hsa_circ_0010889 silencing decreased SATB1 expression. However, miR-590-5p downregulation reversed the inhibitory effects of si-hsa_circ_0010889 to SATB1 expression. When using the post SATB1 overexpression vector, we found that SATB1 expression increased significantly (Figure 4I, 4J) implying that hsa_circ_0010889 promoted SATB1 expression via sponging of miR-590-5p.

### SATB1 overexpression or miR-590-5p inhibition restored glioma cell proliferation and migration post silencing of hsa_circ_0010889

**Figure 5:**
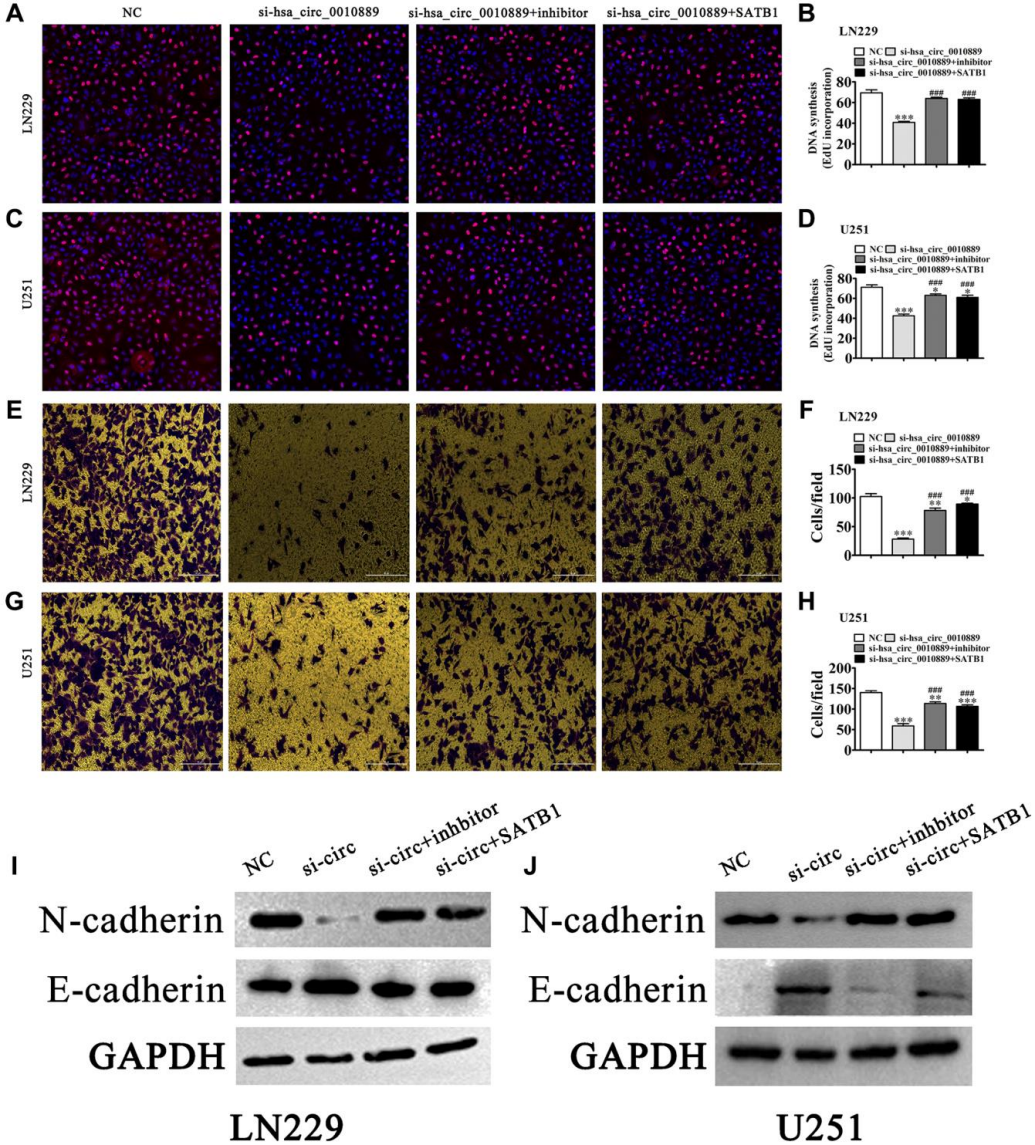
**Overexpression of SATB1 or inhibition of miR-590-5p reversed glioma cell proliferation and migration after silencing hsa_circ_0010889.** (**A**–**D**) EdU detection showing the proliferative ability of LN229 and U251 cells. The data are expressed as the mean ± SD. ^*^P < 0.05, ^***^P < 0.001 vs. NC. ^###^*P* < 0.001 vs. si-hsa_circ_0010889. (**E**–**H**) Transwell detection showing migration of LN229 and U251 cells. The data are expressed as the mean ± SD. ^*^P < 0.05, ^**^P < 0.01, ^***^P < 0.001 vs. NC. ^###^*P* < 0.001 vs. si-hsa_circ_0010889. (**I**, **J**) Western blot results showing the expression of E-cadherin and N-cadherin.

Using EdU (Figure 5A–5D) we found that SATB1 overexpression or miR-590-5p inhibition restored glioma cell proliferation ability in both LN229 and U251 cells post hsa_circ_0010889 silencing (Figure 5C–5E). Transwell assays for the detection of migration showed that SATB1 overexpression or miR-590-5p suppression restored glioma cell migration ability in LN229 and U251 cells post silencing of hsa_circ_0010889 (Figure 5E–5H). Western blot detection show that SATB1 overexpression or miR-590-5p inhibition restored glioma cell EMT-related protein expression post silencing of hsa_circ_0010889 (Figure 5I and 5J).

### Overexpression of SATB1 restored glioma cell proliferation and migration after miR-590-5p overexpression

**Figure 6:**
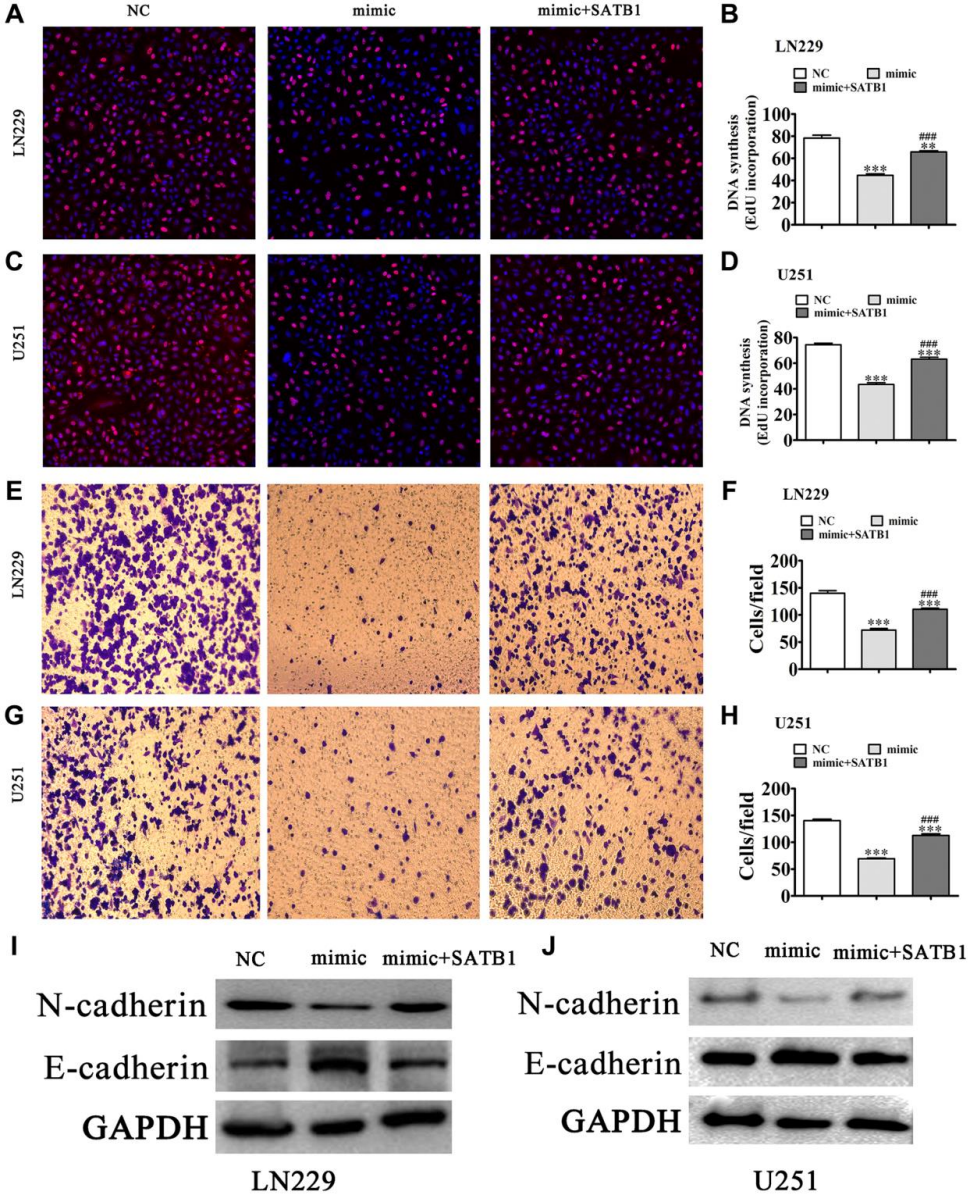
**Overexpression of SATB1 reversed glioma cell proliferation and migration after overexpression of miR-590-5p.** (**A**–**D**) EdU detection showing the proliferative ability of LN229 and U251 cells. The data are expressed as the mean ± SD. ^**^P < 0.01, ^***^P < 0.001 vs. NC. ^###^*P* < 0.001 vs. mimic. (**E**–**H**) Transwell detection showing invasion and migration of LN229 and U251 cells. The data are expressed as the mean ± SD. ^***^P < 0.001 vs. NC. ^###^*P* < 0.001 vs. si-hsa_circ_0010889. (**I**, **J**) Western blot results showing the expression of E-cadherin and N-cadherin.

Again, using EdU (Figure 6A–6D) detection we found that overexpression of SATB1 restored glioma cell proliferation in both LN229 and U251 cells post miR-590-5p overexpression (Figure 5C–5E). Transwell assays also showed that overexpression of SATB1 restored glioma cell migration ability in both U251 and LN229 cells after overexpression of miR-590-5p (Figure 6E–6H). Western blot results showed that SATB1 overexpression restored EMT-related protein expression after miR-590-5p overexpression (Figure 6I and 6J).

## DISCUSSION

Accumulating studies have found that circRNAs are partially responsible for tumorigenesis and glioma progression [10]. |Our present investigation found increased hsa_circ_0010889 expression in glioma tissues and cell lines suggesting that hsa_circ_0010889 is important during glioma progression. Furthermore, hsa_circ_0010889 downregulation inhibited glioma proliferation and invasion but the regulatory mechanisms remain to be elucidated.

Bioinformatics data found that SATB1 and miR-590-5p were downstream targets for hsa_circ_0010889 and this was confirmed by luciferase reporter assay. hsa_circ_0010889 downregulation promoted miR-590-5p expression and importantly previous investigations found that circ_0069718 promotes breast cancer via upregulation of NFIB and targets miR-590-5p directly [11]. miR-590-5p has been found to suppress malignant melanoma cell growth and invasions by targeting Skp2 [12] and downregulation of circ-PITHD1 can inhibit colorectal cancer through suppression and the miR-590-5p/HK2 axis [13]. Our present investigation discovered that miR-590-5p overexpression reversed the inhibitory effects of si-hsa_circ_0010889 on glioma proliferation and migration suggesting that hsa_circ_0010889 silencing can inhibit glioma progression by the promotion of miR-590-5p expression.

Further investigations have found that SATB1 is a downstream target for miR-590-5p which was confirmed by luciferase reporter assay. hsa_circ_0010889 downregulation inhibits SATB1 expression but inhibition of miR-590-5p reversed this inhibitory effect with respect to si-hsa_circ_0010889 and SATB1 expression. Previous studies have found that SATB1 has an important function to regulate invasion and metastasis in breast cancer [14]. Furthermore, SATB1 is highly expressed in several cancer cell types [15]. SATB1 has been shown to influence the epithelial-mesenchymal transition (EMT) in lung cancer [16]. The present study also found that hsa_circ_0010889 silencing inhibited EMT-related protein N-cadherin expression and promoted E-cadherin expression. SATB1 overexpression restored EMT-related protein N-cadherin expression and promoted E-cadherin expression. In addition, hsa_circ_0010889 silencing inhibited SATB1 expression, while SATB1 overexpression restored SATB1 levels. SATB1 overexpression also restored migratory ability post hsa_circ_0010889 silencing, suggesting that removing the effect of hsa_circ_0010889 can inhibit glioma progression by promotion miR-590-5p and inhibiting SATB1 expression.

Our investigation provides evidence that hsa_circ_0010889 downregulation can reduce glioma proliferation and invasion via miR-590-5p/SATB1 signaling mediated by the regulation of aerobic glycolysis. Our data has revealed that hsa_circ_0010889 is a promising marker for glioma diagnostics, and which may be extended to the development of drugs targeting hsa_circ_0010889, for the treatment of glioma.
